# Draft genome sequence of *Deinococcus* sp*.* KR-1, a potential strain for palladium-leaching

**DOI:** 10.7150/jgen.42020

**Published:** 2020-02-10

**Authors:** Hironaga Akita, Yuya Itoiri, Akio Kumagai, Noriyo Takeda, Akinori Matsushika, Mamoru Oshiki, Zen-ichiro Kimura

**Affiliations:** 1 Research Institute for Sustainable Chemistry, National Institute of Advanced Industrial Science and Technology (AIST), 3-11-32 Kagamiyama, Higashi-Hiroshima, Hiroshima 739-0046, Japan; 2 Department of Civil and Environmental Engineering, National Institute of Technology, Kure College, 2-2-11 Aga-minami, Kure, Hiroshima, 737-8506, Japan; 3 Graduate School of Advanced Sciences of Matter, Hiroshima University, 1-3-1 Kagamiyama, Higashi-Hiroshima, Hiroshima 739-8530, Japan; 4 Department of Civil Engineering, National Institute of Technology, Nagaoka College, Japan, 888, Nishi-Katagai, Nagaoka, Niigata 940-8532, Japan

**Keywords:** *Deinococcus*, Palladium-leaching, Draft genome sequencing, 16S rRNA, Phylogenetic tree

## Abstract

Strain KR-1 was isolated from pond water collected in Japan. Because this strain was capable of adsorbing palladium particles in sterilized water, strain KR-1 will be a useful biocatalyst for palladium-leaching from metal waste. Here we present a draft genome sequence of *Deinococcus* sp. KR-1, which consists of a total of 7 contigs containing 4,556,772 bp with a GC content of 70.0% and comprises 4,450 predicted coding sequences. Based on the 16S rRNA gene sequence analysis, strain KR-1 was identified as* Deinococcus* sp. KR-1.

## Introduction

Palladium is one of the platinum-group metals and is an important material used in several industries. For example, palladium is utilized in automobile catalytic converters, which are necessary to degrade harmful gases to harmless ones before their discharge into the atmosphere 1. Palladium has a low melting point and is easily cast. For that reason, it is found on electronic circuit boards in many electronic devices, including televisions, computers and mobile phones 2. Although the global demand of palladium is growing with the continuing development of technologies, the recovery rate of palladium from industrial products is low. However, in 2011, only 22% of the palladium was recovered from autocatalysts and jewelry scrap, and then its metal was reused 3. Because electronic circuit boards include several types of rare metals, selective recovery of palladium entails multiple steps of pyro/hydrometallurgical processes, which increases the cost substantially 2. Thus, establishment of a method for selective recovery of palladium would be highly desirable. Here we report the screening and isolation of a palladium-leaching bacterium, strain KR-1. For future in depth genomic studies and industrial applications of this strain, genome sequencing was carried out.

## Materials and Methods

Pond water was collected from Kure city in Hiroshima prefecture, Japan. An R2A plate (pH 7.0; Nihon Pharmaceutical), which contained 9.7 g·L^-1^ K_2_HPO_4_, 3.2 g·L^-1^ KH_2_PO_4_, 0.05 g·L^-1^ peptone, 0.05 g·L^-1^ soluble starch, 0.05 g·L^-1^ yeast extract, 0.05 g·L^-1^glucose, 0.05 g·L^-1^ casamino acids, 0.03 g·L^-1^ sodium pyruvate and 0.005 g·L^-1^ MgSO_4_·7H_2_O was used for isolation. After 1 mL of a 0.0001% (v/v) diluted solution was inoculated onto the plate, the plate was incubated for 1 day at 30°C. Thereafter, several single colonies were re-streaked onto new plates at least three times to obtain pure colonies. After several purified strains were cultivated in R2A medium, the cells were collected by centrifugation and washed twice with sterile water. For palladium biosorption experiments, each washed strain was incubated for 1 h at room temperature in an oversaturated solution of palladium in water. To prepare samples for transmission electron microscopy (TEM), after hydrophilic treatment of copper grids (200 mesh; TED PELLA, USA), experimental samples (50 µL) were placed onto the treated copper grids as previously described 4. TEM analysis was then carried out with a JEM-2011 Transmission Electron Microscope (JEOL, Tokyo, Japan) using an accelerating voltage of 200 kV. Qualitative analysis of metal particles carried out using an Energy Dispersive X-ray Spectrometer (JEOL) revealed that several isolates exhibited palladium biosorption.

We next determined a draft genome sequence for strain KR-1. A sample was prepared for genome sequencing by growing strain KR-1 aerobically overnight at 30°C in R2A medium. Genomic DNA was then extracted from the cultures using a DNeasy Plant Mini Kit (Qiagen, Hilden, Germany) according to the manufacturer's instructions. The concentration and purity of the genomic DNA were measured using a NanoDrop ND-1000 spectrophotometer (Thermo Fisher Scientific, Waltham, MA, USA) and a Quant-iT dsDNA BR assay kit (Invitrogen, Waltham, MA, USA). Sequencing of the genomic DNA was accomplished using MinION (Oxford Nanopore Technologies, Oxford, UK; Flow cell version R9 and rapid sequencing kit) and MiSeq (Illumina, San Diego, CA, USA) sequencers. Default parameters were used for all software used unless otherwise specified. Hybrid *de novo* assembly of the raw data was carried out using Unicycler ver.0.4.7 5. Genome annotation was performed using Prokka ver.1.14.0 6.

16S rRNA gene sequences of the related type strains were compared with reference sequences available in the GenBank/EMBL/DDBJ databases using BLAST. Construction of a maximum-likelihood tree was performed using FastTree2 7, 8.

## Results and Discussion

**Figure 1:**
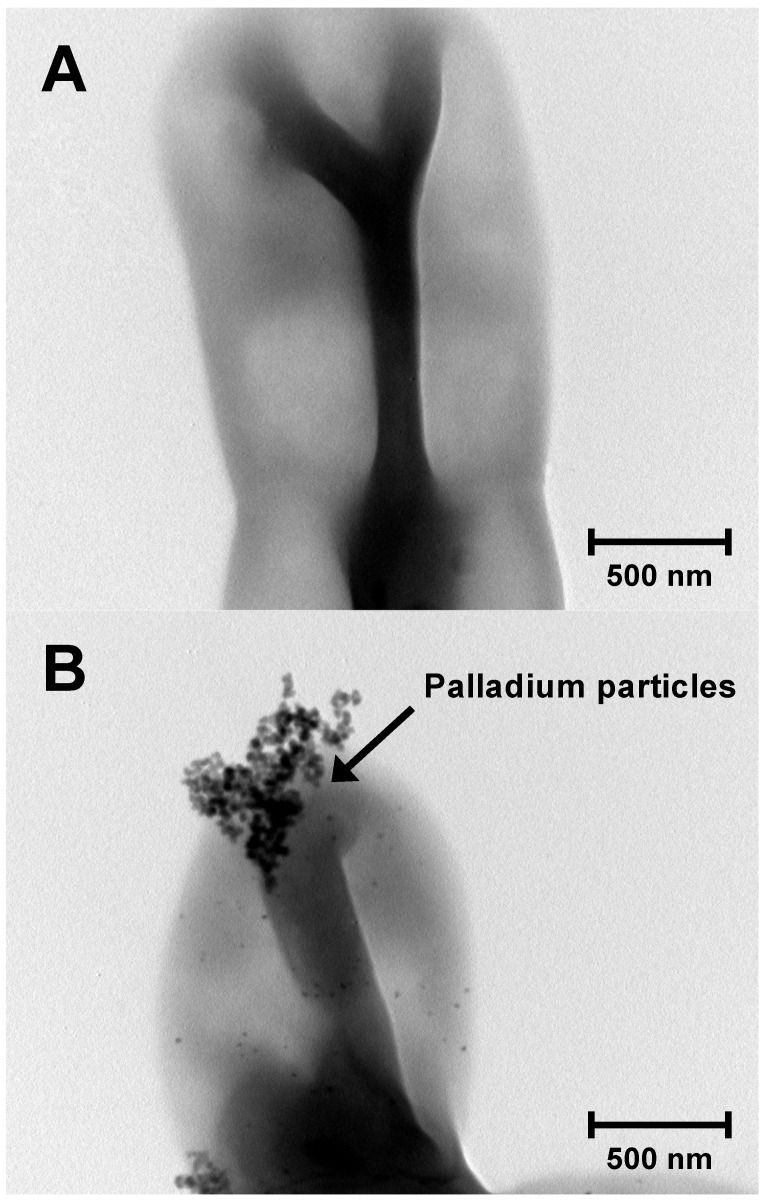
TEM image of strain KR-1 (A) and strain KR-1 with palladium particles (B).

Although various biosorption methods for palladium-leaching are established 9, 10, most of the biosorption methods have not been applied in the industry 2. The reasons are that their culture conditions are intricate and their growth is relatively slow. To establish a practicable biosorption method for palladium-leaching, we propose to apply an oligotroph with a faster growth rate. Because oligotroph grows under conditions of low levels of nutrients, our method may reduce the running cost. To obtain oligotrophic microorganisms, a diluted solution was prepared from pond water and then plated onto R2A plate. After incubation for 1 day at 30°C, several colonies were obtained. Subsequently, we carried out the biosorption experiment of palladium with several isolates. As the result, a few isolates showed palladium biosorption and the highest levels of biosorption were confirmed in strain K-1 (Fig. 1).

We next determined a draft genome sequence for strain KR-1. The raw data from the MinION and Miseq were 46,363 and 2,900,662 reads with 250-coverage. The genome sequence was 4,556,772 bp and the GC content was 70.0%. The assembly generated 7 contigs with an N50 contig size of 3,333,173 bp. Moreover, 4,450 predicted coding sequences were identified. In addition, 53 tRNA genes and 9 rRNA genes were detected using Prokka ver.1.14.0.

To identify the phylogeny of strain KR-1, a maximum likelihood tree based on the 16S rRNA gene sequences was constructed. In the resultant phylogenetic tree, strain KR-1 fell inside the cluster comprising members of the genus *Deinococcus*. Moreover, strain KR-1 exhibited similarities of 99.9 %, 99.9%, 98.5% and 98.8% to its closest relatives, *D. arenae* SA1^T^ 11, *D. actinosclerus* BM2^T^ 12, *D. saudiensis* YIM F302^T^ 13 and *D. soli* ZLM-202^T^ 14. Thus, strain KR-1 was identified as *Deinococcus* sp. KR-1 (strain number: HUT-8138).

When genomic features of *Deinococcus* sp. KR-1, *D. actinosclerus* BM2^T^, *D. actinosclerus* SJTR and *D. soli* ZLM-202^T^ were compared, numbers of tRNA and rRNA as well as the GC content were almost the same (Table 1). On the other hand, the coding sequence number of strain KR-1 was about 1.5 times higher than those of *D. actinosclerus* BM2^T^ and *D. soli* ZLM-202^T^. This result suggested that strain KR-1 may have a unique pathway to grow in oligotrophic media. Although *Desulfovibrio* genus bacteria such as *D. desulfurican D. fructosivorans* and* D. vulgaris* have also the ability to adsorb palladium, the molecular mechanism of palladium-leaching is unclear 10, 15. In this study, we determined the draft genome sequence of *Deinococcus* sp. KR-1, which enables to create the deletion mutants of cell wall protein. To elucidate the molecular mechanism of *Deinococcus* sp. KR-1, we are planning to examine palladium biosorption with several kinds of deletion mutants. The results will be described elsewhere as the next stage of our study.

### Nucleotide Sequence Accession Number

The draft genome sequence of *Deinococcus* sp. KR-1 has been deposited in the DDBJ/EMBL/GeneBank under accession numbers BLBE01000001 to BLBE01000007. The raw sequence reads have been deposited in DDBJ under the BioProject number PRJDB8922 and BioSample number SAMD00192131.

## Figures and Tables

**Figure 2:**
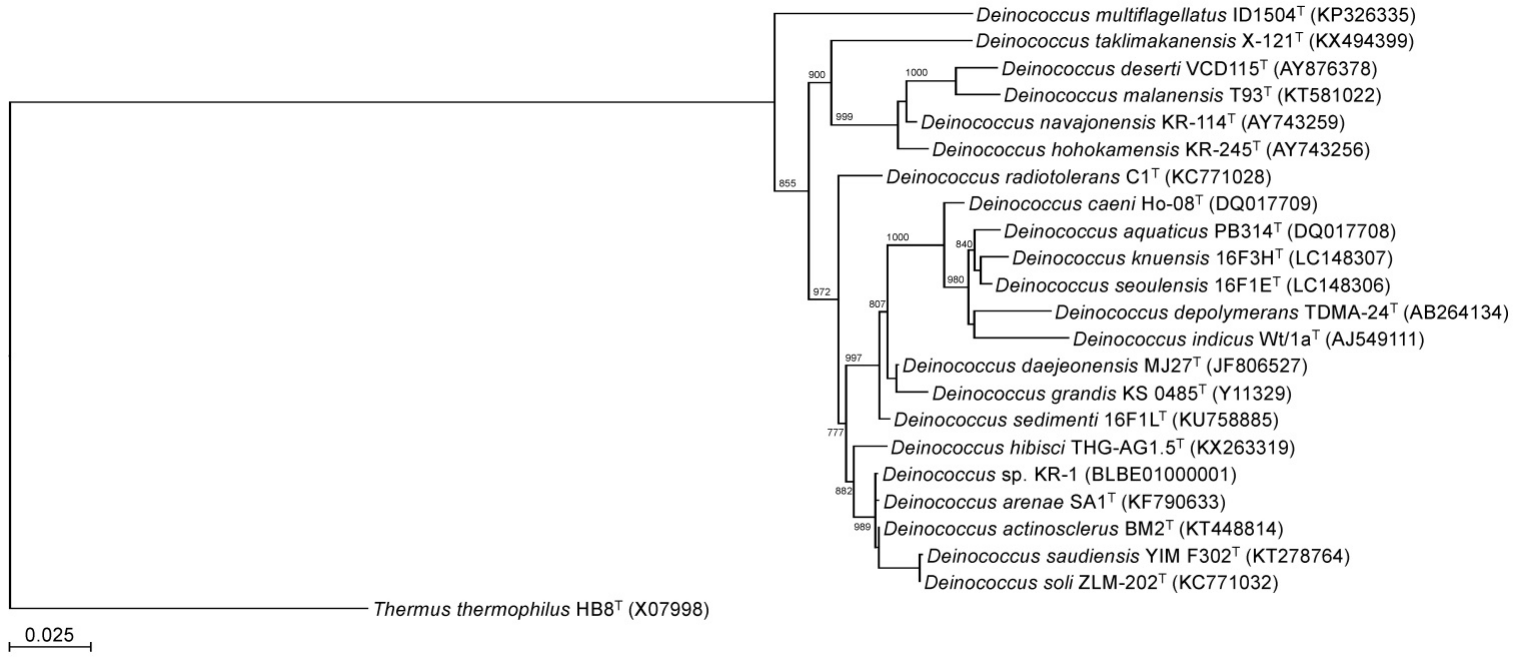
Phylogenetic tree constructed from analysis of 16S rRNA gene sequences showing the relationships between strain KR-1 and its related type strains. The bar indicates a 0.05% nucleotide substitution rate. The tree was rooted using *Thermus thermophilus* HB8^T^ as the outgroup.

**Table 1:** Comparison of general genome features of strain KR-1 with the related type strains.

Strain	*Deinococcus* sp. KR-1 (BLBE01000001-BLBE01000007)	*D. actinosclerus* BM2^T^ (CP013910)	*D. actinosclerus* SJTR (CP029774)	*D. soli* ZLM-202^T^ (CP011389)
Properties				
Genome length (bp)	4,556,772	3,264,334	3,315,586	3,236,984
GC content (%)	70.0	70.6	70.6	70.2
Contig numbers	7	1	1	1
Coding sequence numbers	4,450	3,049	3727	2,944
tRNA	53	49	50	49
rRNA	9	9	12	9
